# Behavioral and Neural Correlates of Communication via Pointing

**DOI:** 10.1371/journal.pone.0017719

**Published:** 2011-03-15

**Authors:** Laurent Cleret de Langavant, Philippe Remy, Iris Trinkler, Joseph McIntyre, Emmanuel Dupoux, Alain Berthoz, Anne-Catherine Bachoud-Lévi

**Affiliations:** 1 INSERM U955, Equipe 1, “Neuropsychologie Interventionnelle,” Institut Mondor de Recherche Biomédicale, Créteil, France; 2 Département d'Etudes Cognitives, ENS, Paris, France; 3 Université Paris-Est, Faculté de Médecine, Créteil, France; 4 AP-HP, CHU Henri Mondor, Service de Neurologie, Créteil, France; 5 URA CNRS-CEA 2210, SHFJ, Orsay, France; 6 LPPA, Collège de France, Paris, France; 7 CESeM UMR 8194, Institut des Neurosciences et de la Cognition, Université Paris Descartes and CNRS, Paris, France; 8 LSCP, Département d'Etudes Cognitives, ENS-EHESS-CNRS, Paris, France; French National Centre for Scientific Research, France

## Abstract

Communicative pointing is a human specific gesture which allows sharing information about a visual item with another person. It sets up a three-way relationship between a subject who points, an addressee and an object. Yet psychophysical and neuroimaging studies have focused on non-communicative pointing, which implies a two-way relationship between a subject and an object without the involvement of an addressee, and makes such gesture comparable to touching or grasping. Thus, experimental data on the communicating function of pointing remain scarce. Here, we examine whether the communicative value of pointing modifies both its behavioral and neural correlates by comparing pointing with or without communication. We found that when healthy participants pointed repeatedly at the same object, the communicative interaction with an addressee induced a spatial reshaping of both the pointing trajectories and the endpoint variability. Our finding supports the hypothesis that a change in reference frame occurs when pointing conveys a communicative intention. In addition, measurement of regional cerebral blood flow using H_2_O^15^ PET-scan showed that pointing when communicating with an addressee activated the right posterior superior temporal sulcus and the right medial prefrontal cortex, in contrast to pointing without communication. Such a right hemisphere network suggests that the communicative value of pointing is related to processes involved in taking another person's perspective. This study brings to light the need for future studies on communicative pointing and its neural correlates by unraveling the three-way relationship between subject, object and an addressee.

## Introduction

The pointing gesture is used to share information about an object with another person [1], [2]. This skill is specific to humans and marks a fundamental step in the development of child social cognition [2], [3], [4], [5], [6]. Although pointing necessarily involves a subject, an object and an addressee to communicate with, previous psychophysical and neuroimaging studies on the pointing gesture have focused on the relationship between a subject and an object. However, the addressee is a crucial factor in communicative pointing.

Pointing, referred to here as communicative pointing (CP), is acquired at the end of the first year of life and is tightly linked to one's ability to perceive and monitor the addressee's attention onto the target [7], [8]. Children typically gaze at the addressee before pointing, to engage his or her attention, and after pointing, to check for their success in sharing attention upon the target [7]. Infants point more often when their addressee can see them [9] and when the addressee can see the target [10] than when he or she cannot, thereby showing that the infant can integrate the position and the perspective of the addressee [4], [11], [12]. Critically, both the absence of acquisition of pointing [6] and the lack of monitoring of another person's attention toward an object of interest [12] are key diagnostic features of developmental deficits of communication such as autism [4], [6], [13], [14]. The absence of or the delay in the emergence of pointing behavior in autistic spectrum conditions led to the hypothesis of a deficit in a ‘Shared Attention Mechanism’ (SAM) [15], [16]. In this model, two representations are combined: a first representation that takes into account the relation between the subject and the object (‘I see X’, first person's perspective), and a second representation that specifies the relationship of another agent on the same object (‘He/she sees X’, third person's perspective). From these two dyadic representations, the SAM could form a triadic representation supporting the three-way relationship for CP (‘He/she sees that I see X’). Patients with autism would build correctly dyadic representations but would be impaired in combining them within the SAM [15], [16]. This hypothesis has brought considerable attention in the domain of social cognition; however, experimental evidence has yet to be provided.

A rather unexpected contribution to the SAM hypothesis comes from studies of acquired pointing deficits in brain lesioned adults. Indeed, patients with heterotopagnosia cannot point at another person's body parts, whereas they can grasp or touch them [17], [18]. Instead of pointing at another person's body, they systematically point at their own body, in what we called ‘self-referencing behavior’ [17], [18], [19]. Apart from the target specificity for body parts, this phenomena indicates that the patients' ability to form three-way relationships used for communication through pointing is impaired, in contrast with their ability to form two-way relationships used for grasping and touching [19]. In addition, the fact they self-refer suggests that they only rely on spatial coding with respect to their own body (egocentric representations) while lacking other types of spatial reference linked to the addressee. This pointing-versus-grasping dissociation in heterotopagnosia is a critical argument that underlines the specific communicative value of pointing compared to other arm gestures. It has not yet been observed in other pointing deficits such as autotopagnosia, the deficit in pointing at one's own body or at any body parts [20]. Accordingly, in this latter case the impairment of a body representation is usually suspected [21] rather than the disruption of the communicative function of pointing that we hypothesize for heterotopagnosia [19] (but see [18] for an alternative view). Therefore, we capitalize on the pointing-versus-grasping dissociation of heterotopagnosia to postulate the existence of dedicated cognitive and brain correlates related to communication with an addressee via pointing.

Pointing, grasping and touching are all visuo-motor gestures that require complex motor planning to be achieved. Spatio-temporal parameters of these movements are coded along different frames of reference, either egocentric (with respect to one's own body) or allocentric (with respect to the environment), the existence of which have already been demonstrated by psychophysical studies [22], [23], [24], [25], [26]. Yet, these studies focused on the relation between a subject and an object, leaving aside any communication with an addressee. As a consequence, these experiments explored non-communicative pointing (NCP), which is conceptually similar to visuo-motor actions such as grasping or touching and distinct from CP. Thus, the specificity of CP, as revealed in heterotopagnosia, has not been experimentally explored. Indeed, the transition from the two-way relationship in grasping or touching to the three-way relationship of CP presumably induces a modification in the frames of reference in relation to the addressee [19]. Such spatial coding might be captured by using a dedicated experimental set-up.

Similarly, the neural basis of the communicative value of pointing cannot be inferred from previous neuroimaging studies on NCP [27], [28], [29], [30]. The hypothetical SAM that creates triadic representations for CP was suspected to be based in the superior temporal sulcus [15] because this region is sensitive to the orientation of another individual's visual perspective. This view is supported by single-cell recordings in non-human primates [31], [32], [33], by neuroimaging studies in humans [34], [35], [36] and by individuals with autism who show anatomical and functional anomalies in the STS region [37]. Yet, it is not clear why this region should combine two perspectives, with respect to one's self and with respect to the other, because it mostly refers to a third-person perspective. The communicative value of CP could also rely on brain areas that are impaired in patients with long-lasting heterotopagnosia, namely the left posterior parietal cortex and the insula [19]. However, because in these patients the deficit combines a particular kind of target, the body of others, with the specific task of pointing, heterotopagnosia might relate to the combined deficit of both a body representation and the pointing process itself.

**Figure 1:**
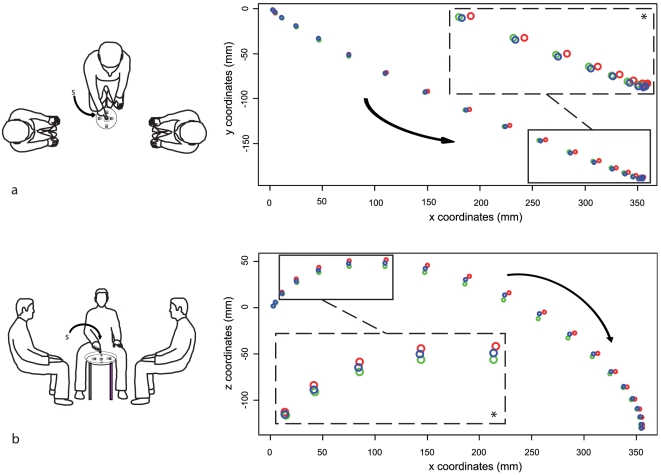
Setting and pointing trajectories in Experiment 1. Top (a) and side-view (b) of the psychophysics experiment (Experiment 1) showing left and right-addressees facing each other and located to each side of the participant, and objects located on the table in front of them. c) and d): Mean trajectories of pointing movements for horizontal (c) and frontal (d) planes in the three conditions (red  =  left CP; blue  =  right CP; green  =  NCP). Arrows indicate the direction of the movement and the letter “s” the starting position. Coordinates x, y, z are in mm. Rectangle windows highlight points that are statistically different across conditions. Enlarged views of these points are indicated in dotted-line rectangle windows.

Here, we examine whether CP differs from NCP in terms of reference frames when an object is used as a target, leaving aside the question of the specificity of the human body. To do this, we use a combination of behavioral and imaging (PET-scan) techniques in our study of CP and NCP in healthy participants. Previous studies showed that endpoint variability for repeated pointing gestures can reveal the reference frames used for movement planning [25], [38], [39], [40]. Thus, in a first psychophysical experiment, using a 3D tracking device, we checked if kinematics and endpoint variability for repeated pointing gestures, addressed or not to another person, would change according to the communicative interaction with an addressee. During CP conditions, the participant pointed at a target in order to have it named by an addressee. He (she) called one out of two addressees, then pointed at an object and then the designated addressee named the indicated target (Figures 1a and 1b). During the NCP condition, the participant pointed at an object without any interaction with an addressee even though the addressee was still present. We predicted that gestures in CP and in NCP would differ and reveal specific modifications of reference frames in CP.

**Figure 2:**
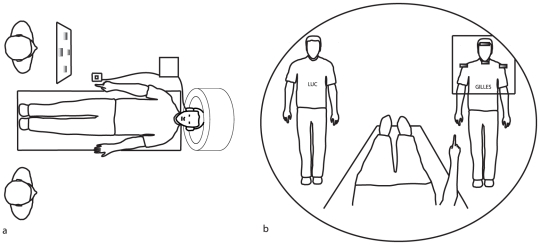
Setting of the PET study in Experiment 2. Top view (a) and participant's view (b) of the setting are provided. Artifacts to be pointed at are fixated on a vertical Plexiglas pane. The participant can see from the scanner bed both the addressees and the four artifacts to be pointed at.

Then, we adapted this paradigm to a PET-scan study in order to determine the neural correlates for the communicative value of CP. Participants communicated with addressees who faced them during scanning session (Figure 2). During the NCP condition, both addressees kept their eyes closed at hearing their name and the participant pointed at an object. During the CP condition, the participant called on one of the two addressees to open his eyes, and then pointed at an object. After the end of each pointing gesture, the participant was told the name of the target to which he had pointed, either from the addressee in the CP condition or from the computer in the NCP condition. This experiment allowed the comparison between CP and NCP at the neural level. We expected to reveal the brain network that accounts for the communicative interaction with an addressee while pointing.

## Methods

### Experiment 1: Psychophysical study of CP and NCP

#### 1) Participants

Ten right-handed [41] healthy volunteers (8 women, 2 men), aged 28.1±8.6 years, were tested. Participants' mean education level was 15.6 years after primary school and none had a history of neurological disease or treatment. The protocol and these experiments were approved by the ethical committee of Henri Mondor Hospital. All participants gave their written informed consent. They were not informed of the background hypothesis.

#### 2) Procedure and apparatus

The participant sat on a 45-cm-high straight-back chair facing a 50-cm-high table. Two addressees were seated symmetrically at a distance of 1.3 m, flanking the participant to the left and right (see Figure 1). They wore T-shirts on which was written the name they were attributed for the experiment (Gilles, Maud, Luc, Jeanne).

Five objects with similar dimensions (battery 4×2 cm, lighter 4×1.5 cm, salt cellar (4×3 cm), eraser (4×2 cm), and a small round opaque glass, 3 cm in diameter and 3 cm in height) were placed on the recessed surface of a table, 10 cm apart, in a cross arrangement and covered by a Plexiglas pane (see Figure 1). The axes of the peripheral objects were aligned with the branches of the cross arrangement of the array. The glass was placed at the centre of the array, its top being 2 cm away from the Plexiglas pane. Only the circular glass was isotropically situated in this setting because of its central position and because of its round shape that lacked any intrinsic axis [26]. Therefore, this object was used as the principal target for this study. The four other items were used as distractors to entertain the attention and the communicative interaction of the participants over the course of the experiment.

The participant wore headphones in order to hear the instructions for pointing. Therefore, he/she was the only one to know which object he/she had to point at. His/Her right forearm lay on a 65-cm-high armrest, with the index finger holding down an answer button. He/She released the button when pointing, thus providing the onset on the gesture. Movements were recorded until the fingertip touched the Plexiglas pane.

After training, each participant pointed 20 times toward each of the 5 possible target objects in 3 conditions, for a total of 300 trials. Conditions differed with respect to the communicative intention: pointing was either addressed to the left or right person (Left CP and Right CP), or not addressing anybody (NCP). Trials were grouped into 30 blocks (10 blocks for each condition, each item being pointed to twice within each block). Before each block, the participant was instructed through headphones whether pointing would be addressed to another person or to nobody. Therefore, at the beginning of a new block the participant called aloud one of the two addressees or said ‘nobody’ according to the condition. Within a block, the instructions indicating which target to point to were randomized. Each instruction was of the type “show Gilles the eraser” in CP conditions, or “show the saltcellar” in NCP condition. As soon as they understood the name of the target, then he/she was free to point at it. In the CP conditions, the designated addressee said aloud the name of the indicated object and the participant acknowledged a correct or erroneous answer by a nod or a shake of the head, as appropriate. In the NCP conditions, the participant had no feedback from the addressee. After each trial, the participant pushed down the answer button again, in order to hear the next instruction.

#### 3) Data Acquisition

Both the answer button and headphones were connected to a computer. Reaction times were recorded from the onset of the target name until the release of the answer button, using the Expe software [42], with a temporal resolution of 1 millisecond. The kinematics of the movements were recorded by a CODA tracking system (Charnwood Dynamics) fixed at a height of 2 m in front of and to the right of the participant. This tracking system records the 3D trajectories of active LED markers with a spatial resolution of 0.1 mm at a frequency of 400 Hz. One LED marker (LED1) was attached to the right index fingertip of the participant. A second marker (LED2) was turned off and on by the answer button, allowing kinematic data acquisition to be synchronized to the start of the participant's response. Two additional LEDs (LED3 and LED4) were attached to the left and right shoulders of the participant, in order to measure the movement of his or her trunk during the gestures.

The coordinates of the right index finger pushing down the button at the starting position were chosen as the origin for spatial coordinates. The x-axis was defined as the horizontal line parallel to the line linking the shoulders of the participant, and was oriented from his/her right to his/her left. The y-axis was the horizontal line perpendicular to x-axis and oriented from front to back of the participant. The z-axis was the vertical line perpendicular to the previous axes and oriented from bottom to up.

#### 4) Data analysis

a) Treatment of the coordinates of the movement from CODA tracking system.

First, an exponential filter was used for each movement: for two successive points (i) and (i- x1) with coordinates (x_i_, y_i_, z_i_) and (x_i-1_, y_i-1_, z_i-1_) respectively, the filter xx_i_ = x_i-1_+(x_i_–x_i-1_)×0.1 was applied. The same filter was used in the three dimensions of Cartesian space. Instantaneous tangential velocity was calculated for each point in the sample:
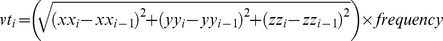



The beginning of each movement was determined as the first point where velocity reached 5% of the maximal velocity. Similarly the end of each movement corresponded to the first point at which velocity decreased below 5% of the maximal velocity. Movements with several maximal velocity peaks corresponding to errors in the selection of the target were considered invalid and rejected.

b) Reaction Times and other temporal parameters.

Reaction time (*RT*) data provided by the Expe software were corrected by means of the movement data provided by the CODA system. For each movement, the small temporal gap between the release of the answer button ( =  the extinction of LED2) and the effective beginning of the movement was added to the reaction time provided by Expe to give the corrected reaction time (*cRT*). The duration (*dur*) of the movement is defined by the time when instantaneous velocity exceeded 5% of the maximal velocity (*maxvel*). Time to peak of maximal velocity (*ttp*) is defined by the time between moment of first reaching 5% of *maxvel* and *ttp*. The *distance* is the Cartesian distance between the initial position of the finger to its end position. Mean velocity (*meanvel*) is defined by the ratio *distance/dur*. These temporal parameters were subjected to ANOVA analysis using condition (Left CP, Right CP and NCP) as the independent variable for within-participant and within-item comparisons.

c) Spatial parameters.

*Trajectories of pointing.* For each pointing gesture, a sample of 20 points along the trajectory were isolated at equivalent temporal intervals (duration of the interval  =  duration of the movement/20). Coordinates of each point (x, y, z) were submitted to an ANOVA with conditions as independent variables for within-participant analysis. For each condition, we calculated the coordinates of the 20 average points of the trajectories in order to offer a graphical representation of the trajectories.

*Endpoint variability.* Repeated gestures towards the same object led to a slight variance in endpoint position around the target location [23]. Analysis of endpoint variability focused on the round glass at the centre of the target array, for which the allocentric coordinates evoked by the surrounding environment or the target shape itself did not bias the gesture [26]. Similar analysis was nevertheless run for each of the peripheral objects.

In order to exclude outliers in fingertip endpoints, an analysis of Cook's distance [43] for each point, for each participant and each condition was performed, using the R software [44]. This analysis permits the measure of the influence of each endpoint on the overall distribution of endpoints. A 95% confidence interval analysis was obtained by excluding outliers defined by Cook's distance that exceeded 0.25. We calculated 2×2 covariance matrices for the x and y coordinates of endpoints for each participant and condition using R, resulting in 30 matrices (3 conditions × 10 participants). Matrices were normalized for size variations by dividing each individual matrix by its first eigenvalue. A graphical representation of a 2×2 covariance matrix is an ellipse defined by its long and short axes. The orientation and size of the axes of the ellipse are defined by eigenvectors and eigenvalues of the matrix. In order to observe the general gesture behavior across participants, we computed the average matrix and the corresponding average tolerance ellipse for each of the 3 conditions (Left CP, Right CP and NCP).

To test statistically if the orientation of the ellipses was different between two conditions, we ran Monte Carlo simulations [45]. For each subject *s*, we compared the orientation of the tolerance ellipse computed from the sample S_a_ of *n* endpoints performed in condition *a* with the orientation of the tolerance ellipse computed from the sample S_b_ of *m* endpoints performed in condition *b* (n could be different from m due to outlier rejection from one sample or the other). In order to test the null hypothesis that samples S_a_ and S_b_ were drawn from the same population, we created computed pairs of samples S_a_^sim^ (n points) and S_b_^sim^ (m points) by drawing randomly, with replacement, from the union of the two data sets (S_a_ ∪ S_b_). Ten thousand simulations were run for all subjects and for each comparison (Left CP versus Right CP, Left CP versus NCP, and Right CP versus NCP). We calculated the average tolerance ellipse for each simulated sample in each pair and calculated how many simulations (N) gave the same or greater difference of angle as that observed between the true empirical samples S_a_ and S_b_. This gave the probability measure p = N/10000 that the results could be attributed to chance alone.

*Initial trunk position.* The mean coordinates of LED3 and LED4 allowed the measure of the trunk orientation of the subject at the beginning of the movement. This analysis was performed to check if any differences in endpoint variability between the three conditions could be due to differences in trunk orientation when addressing somebody located to the left or to the right, even before the pointing gesture began. The angle of the vector linking the shoulders and the x axis was computed in the three conditions across the subjects. Monte Carlo simulations were computed to test the significance of observed difference of such angles between two conditions.

### Experiment 2: PET activation study of CP and NCP

#### 1) Participants

Ten male and right-handed healthy volunteers, 25.8±5.2 year-old (mean education level of 14.8 years after primary school) who did not participate in Experiment 1, were enrolled in this experiment. Only male participants were chosen because of the potentially noxious effects of the PET scan technique for fetuses in case of unknown pregnancy in women. They had a no history of neurological disease or treatment. The protocol was approved by the ethical committee of Henri Mondor. All signed the informed consent.

#### 2) Procedure and apparatus

The general procedure and apparatus were similar to the ones used for Experiment 1 except for a few adaptations required by the imaging procedure (Figure 2). PET-scan was chosen rather than fMRI, in order to allow the scanned participant to see living addressees in front of him during the whole experiment.

The participant was instructed not to move except for the execution of the tasks. His head was immobilized on the scanner bed with an individually fitted, rigid, thermoplastic mask. Prior to scanning, a small plastic catheter was placed in the cubital vein of the participant's left arm for injection of the radioisotope. The participant's right arm rested on a shelf with the right index finger positioned near an answer button. The participant wore headphones to listen to the instructions. Two addressees were standing on stools in front of him, on either side of the scanner bed (Figure 2). They wore T-shirts written with their name: “Gilles” on the right and “Luc” on the left.

Four target objects (battery, eraser, left lighter, right lighter) were fixed on a vertical Plexiglas pane, on the right side of the scanner bed. These items were located between the right arm of the participant and the addressee “Gilles”. Therefore during the whole experiment, the participant could see “Luc” on his left and “Gilles” on his right behind the four objects.

Each auditory stimulus was composed of three elements: instruction for calling the name of a person (e.g. “call Gilles” or “call Luc”), instruction for the task (e.g. “show Gilles” or “show”) and the target (e.g. “the eraser”). The participant had to point with his right index finger at the target item.

After training, 288 instructions were given to each participant. Instructions were grouped into 12 blocks resulting in 3 blocks of 24 trials for each of the above 4 conditions. Before each block, the participant heard an explanation about the task to be performed. Before each trial, the participant was instructed to push down the answer button in order to hear the next instruction. The button had to be held down until the beginning of the next pointing gesture. Within each block the trials were presented in random order.

Measures of regional cerebral blood flow (rCBF) were analyzed under two conditions: 1) pointing at an object without any intention to communicate (NCP); 2) pointing at an object with the intention to communicate with an addressee (CP). Before the beginning of each block, both potential addressees Gilles and Luc knew what they should do, irrespective of what the participant said. In the NCP condition the participant heard for example “call Gilles”. He then had to say aloud “Gilles” while gazing at him. In this condition, both Gilles and Luc knew that they should not react nor open their eyes at hearing their name. Then the participant heard, for example “show the eraser”. He lifted his right finger from the button to execute the pointing gesture. After pointing, the participant heard again the name of the pointed target, i.e. “the eraser”, presented by the computer through his headphones. The participant stopped pointing to the target and pushed down again the button to hear another instruction. In the CP condition, the participant heard, for example, “call Gilles”. He then had to call aloud “Gilles” while gazing at him. In this condition, both addressees opened their eyes at hearing their name. Then the participant heard, for example, “show Gilles the eraser”. The participant lifted the right finger from the button to execute his gesture. The addressee “Gilles” said aloud the name of the indicated object. Subsequently, the participant pushed down the button again to hear the instruction for the next trial.

The whole experiment was video-recorded to control whether participants understood the instructions and acted accordingly.

#### 3) Data Acquisition

Reaction times were measured as in Experiment 1 by Expe software. Positron emission tomography measurements were performed using a tomograph that allowed the 3-dimensional acquisition of 63 transaxial slices (EXACT-HR+; CTI-Siemens, Knoxville, Tennessee). Spatial resolution was 4.5 mm and 4.1 mm in the transaxial and axial directions, respectively. Regional cerebral blood flow images were acquired 10 minutes apart, for 80 seconds after the injection of 8 mCi of H_2_^15^O. Each image acquisition corresponded to several gestures in one block and movements started 40 seconds before image acquisition. Each of the four conditions was performed three times, giving a total of 12 scans per participant.

#### 4) Data analysis

Reaction times were analyzed with ANOVAs using condition (CP versus NCP) as the independent variable for the within-participant and within-item analysis. Image analysis was performed using statistical parametric mapping (SPM2; http://www.fil.ion.ucl.ac.uk/spm/). The 12 images obtained in each participant were realigned, normalized to Montreal Neurological Institute (MNI) space, and smoothed with a Gaussian kernel of 8 mm full width at half maximum. We contrasted the images obtained during CP and NCP. P values were set at 0.05 FDR (False Discovery Rate) corrected for multiple comparisons in a whole brain analysis, which yielded a minimal cluster size of 5 voxels.

## Results

### Experiment 1: Psychophysical study

Participants made less than 0.1% errors (showing for example the eraser instead of the lighter) in their gestures, without any difference between conditions. Altogether, addressees made 3 errors (0.04%) in identifying which target was designated during the whole experiment.

Analyses of non-corrected and corrected reaction times yielded similar results. Reaction times and other temporal parameters of the movement did not differ significantly for CP and NCP (p>0.05) (Table S1).

However, the analysis of spatial parameters revealed a significant shift of the direction of pointing toward the Left and Right addressees, for Left CP and Right CP trajectories respectively. This shift was observed at the end of the movement along the x-axis (p<0.05; Figure 1c and Table S2). Furthermore, a slightly higher ascent was observed for the trajectories of CP compared to NCP along the vertical z-axis at the beginning of the movement (p<0.05; Figure 1d and Table S3). There was no difference, on average, between the trajectories of the three conditions along the y-axis (p>0.05).

#### Endpoint variability

**Figure 3:**
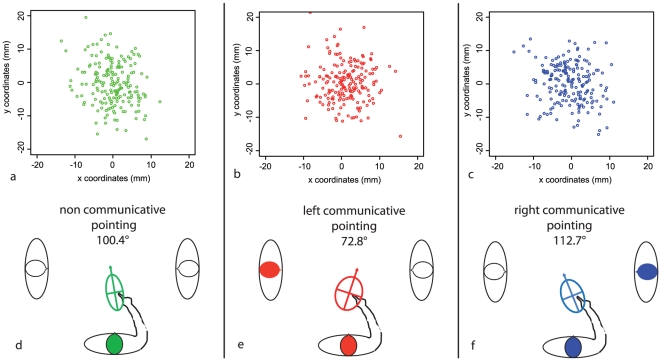
Endpoint variability in Experiment 1. Endpoint variability in each pointing condition is represented as tolerance ellipses in the three conditions. Endpoints for the three conditions: a) NCP (in green); b) Left CP (in red); c) Right CP (in blue). The orientation of the 2D-ellipse varies across conditions. The angle is measured between the main axis of the ellipse and the frontal plane of the participant: d) NCP; e) Left CP; f) Right CP.

Four percent of outliers were excluded from the analysis (see methods above). The average normalized tolerance ellipses of endpoint variability for each condition for the central target are shown in Figure 3. The long axis of the tolerance ellipse formed a 100.4° angle with the frontal plane in NCP, whereas it was oriented toward the right (72.8°) for pointing to the left addressee (Left CP) and toward the left (112.7°) for pointing to the right addressee (Right CP). The difference in the orientation between Left CP and Right CP (39.9°, p = 0.022) and between Left CP and NCP (27.6°, p = 0.025) were statistically significant according to Monte Carlo simulations. The difference in orientation between Right CP and NCP was not significant (12.3°, p>0.05) (Figure 3). Ellipses for the peripheral targets were roughly oriented along the axes of these items (Figure S1) [26] and did not differ significantly, as expected (p>0.05).

#### Initial trunk orientation

The orientation of the subjects' trunk just prior to the pointing movement was similar in each condition (Figure S2; p>0.05).

### Experiment 2: PET study

As in previous experiment, addressees perfectly acknowledged targets of pointing (99.99%). Reaction times were similar for CP and NCP: 993.2±48 ms and 959.1±44.7 ms respectively (F (1, 9) = 2.5; p>0.05). Post-hoc analysis showed that reaction times were shorter for targets located on the midline of the array than for those to either side (821.9±42.5 ms and 1126.6±48.9 ms, respectively; F (1, 9) = 297.3; p<0.001; see Figure 2).

#### Imaging data

**Figure 4:**
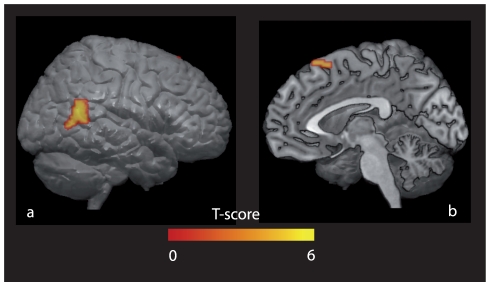
Brain correlates of communication via pointing. Brain areas showing greater activation for communicative (CP) versus non-communicative (NCP) pointing are plotted onto 3D render MNI templates. a) right posterior STS at the temporo-parietal junction (lateral view, right hemisphere), b) right pre-SMA (medial view, right hemisphere) (FDR corrected, p<0.05).

Compared to NCP, CP was associated with an increase of rCBF in two large clusters and a smaller one: the right middle temporal gyrus at the posterior part of the right superior temporal sulcus (STS), close to the temporo-parietal junction, the right medial superior frontal gyrus corresponding to the pre-Supplementary Motor Area (pre-SMA) and the left precuneus (Table 1 & Figure 4). The reverse comparison (NCP – CP) did not yield any significant increase of rCBF for the chosen threshold of analysis.

**Table 1:** Brain activations for the contrast CP – NCP in Experiment 2.

Brain area (number of voxels)	Side	BA	T	MNI coordinates		
				x	y	z
Middle temporal (318)	R	21, 22	6.48	58	−52	18
		37	5.30	56	−60	4
		37	4.06	46	−58	10
Medial superior frontal (26)	R	8	4.40	2	28	62
		6	4.19	4	18	60
Precuneus (5)	L	-	4.13	−2	−52	44

Threshold of analysis: T = 3.89; p _FDR corrected_<0.05, minimal number of voxels = 5.

## Discussion

The comparison of CP and NCP in this study reveals that the communicative interaction with an addressee yields not only behavioral but also neural modifications. We show that adding social value to the pointing gesture has spatial consequences: both the hand trajectories and endpoint variability were influenced by the interaction with and the location of the addressee. This indicates that reference frames for the movement of CP are different from those of NCP. Furthermore, our PET study indicates that CP recruits a right hemisphere network including the posterior STS and the pre-SMA when compared to NCP. We discuss these behavioral and neural modifications with regard to the nature of the communicative interaction with an addressee that is required for CP but not for NCP. These results support the activation of a reference frame linked to the addressee's perspective during CP.

The experimental setting of Experiment 1 rules out the possibility that any low-level perceptual difference between CP and NCP conditions could account for the results. The same hand, same starting position, same presence and location of the addressees, and same targets were used for the whole experiment. In addition, the center-surround placement of the objects was directionally neutral for the central target used in the main analysis. Reaction times were similar for CP and NCP in both the psychophysics and the PET experiment, although participants were slower, overall, in the PET study, presumably due to the lateralization of two of the targets and to the participant's supine position. In contrast, spatial differences were found in trajectories (Figure 1) and in endpoint variability (Figure 3) according to the communicative value of pointing in Experiment 1. Both of these changed depending on who (and thus where) was the addressee of the pointing gesture. The analysis of endpoint variance ellipses gives an insight into the reference frames used for the three conditions [21], [22], [23]. The orientation of the long axis of the ellipse towards the participant's body and moving arm in the NCP condition suggests the use of an egocentric reference frame, as already reported in goal-directed movements [24]. However, in CP conditions, the ellipses were tilted away from the line of sight of the addressee. The orientation of the ellipses measured in Left CP differed from those measured during NCP (Figure 3), thus providing evidence for a change in reference frames between CP and NCP. In addition, the observed difference between Left CP and Right CP ellipses further suggests that such modifications during CP depend on the addressee's location (Figure 3) and are thus specifically linked to the communication between the participant and the chosen addressee.

One might ask why there was a difference between Left CP and NCP, but not between Right CP and NCP. NCP is known to yield an orientation of tolerance ellipses toward the participant's body (indicating an egocentric reference frame), but rotated slightly toward the arm used to perform the pointing gesture and toward the starting point of the movement [25]. Therefore, the fact that both Right CP and NCP induce a shift of the tolerance ellipse toward the right means that it is easier to detect a difference between Left CP and NCP than between Right CP and NCP.

The changes in CP ellipses may reflect additional steps for non-verbal communication. Indeed, directing another person's attention is a prerequisite for addressing him or her during pointing. This has been stressed for both typical and atypical development in children [4], [10], [11], [12], [15]; the involved processes concern several components such as shifting of attention toward the addressee, capturing the attention of the addressee, taking the perspective of the addressee, and finally interacting with him or her about the target. Undoubtedly, each individual component participates in the spatial modulation of both trajectories and ellipses that we observed. In addition, the attention of the pointing participant could be attracted by the objects surrounding the target or by the persons flanking the participant during the experiment. Such an attraction can cause variability of the movement trajectories [46] or of the endpoints [47]. However, as only the instructions, but not the environment, changed between CP and NCP, the modification of the pointing behavior must be due to either the interaction between the participant and the addressee or to the integration of the perspective of the addressee on the target by the participant. The deviation of the trajectories at the end - and not the beginning - of the movement along x-axis (Figure 1c) likely reflects neural processing related to the addressee's attention onto the object, rather than an initial shift of the attention of the participant toward the addressee. In accord with this reasoning, the initial orientation of the subjects' trunk before the movement was similar in all three conditions (Figure S2), indicating that the final differences in trajectories and endpoint variability are not the consequence of a biased orientation of the trunk.

Altogether, our results indicate that the pointing participant models the addressee's perspective onto the target while communicating [15], [16], [19] and thereby makes the pointing gesture more intelligible for the addressee. Indeed, both the lateral deviation of the CP trajectories and the reshaping of the CP ellipses help the addressee to discriminate the one designated target amongst the five closely-spaced objects. Such higher order contextual information, like penalty/reward expectations, is known to influence the shaping of endpoint variability in healthy participants [48]. Here, addressees responded correctly from the very beginning of the test trials; thus, the reshaping of endpoints variability comes from an *a priori* prediction of the addressee's ability to discriminate the target rather than from an *a posteriori* experience of success and failure. Finally, the reshaping of the CP ellipses not only integrates the egocentric reference frames oriented towards the participant's body, but also another reference frame related to the location of the addressee. Such spatial coding for allocentric reference frames has already been invoked to describe the ability to take the perspective of another partner during social interaction [49], [50]. However, here we suggest that the reference frame for CP is built with respect to the addressee's body and perspective, rather than with respect to an object. These CP ellipses therefore manifest what we have called the heterocentric reference frames linked to the addressee [19] and different from allocentric reference frames which are not specific to the communicative context [26].

In the PET study, the communicative aspect of CP recruited a right brain network (Figure 4). The activation of the pSTS (Table 1) has previously been associated with various aspects of social behavior. It is involved in social perception (identifying biological motion [51], evaluating human gaze orientation [34], assessing another's visual attention for an object [36], etc., see [37], [52] for reviews) or in more complex social abilities (detecting intentional actions performed by someone else [53], interpreting and imitating the actions of others [54], or representing the mental states of others [55]). Right-lateralized activations of the STS have been associated with tasks related to another person compared to the self [36], [53], [56], [57], [58], the representation of a person in space [59] and taking another person's visual perspective [60], [61]. Accordingly, the electric stimulation of the right temporo-parietal junction can elicit the experience of seeing one's own body from an external perspective [62]. In addition, the closely located right inferior parietal cortex is activated when patients misattribute the agency of an action to another person, as is the case in delusions of alien control in schizophrenia [63], [64], [65]. Our findings showing the right pSTS activation suggests that the communicative value of CP involves a specific processing of the addressee's perspective. Consistently, the SAM was supposedly located in the STS region [15].

Another important finding is the activation of the pre-SMA during CP. Note that the right sidedness of this activation should be examined with caution given the uncertainty of anatomical left/right lateralization for areas close to the sagittal plane in PET studies. The pre-SMA has been associated with cognitive control in tasks that require the learning of motor sequences, the shifting of attention towards a selected target and the expectation of the outcome of a given assay (for a recent review, see [66]). Such cognitive control might be incurred in many aspects of our CP task during the PET study, where participants achieved a sequence of gaze alternations between an addressee and a target, and expected an answer from the addressee after pointing. Previous studies found that the pre-SMA is more active when the movement is internally driven and less active when the movement is a reaction to an external stimulus [67], [68], [69]. Therefore, such pre-SMA activation suggests that the modification of brain metabolism observed for CP is not the consequence of the detection of external social cues such as the opening eyes of the addressee or their voice. One possible explanation consistent with the data is that recruiting the pre-SMA during CP reflects additional cognitive control processes with respect to attention shifting, outcome expectation, monitoring of the sources of information (the computer or the addressee). Finally, such a pre-SMA region might play an important role in the development of CP because it was shown that brain-evoked potentials in frontal regions in 14-month-old toddlers correlate with later performance in CP at 18 months [70].

In conclusion, genuine CP requires not only the presence of another human, but also that this human enters a communicative relationship with the pointing subject. We provide evidence that both the participant's behavior and brain activity during CP are sensitive to the relationship with a designated addressee, and not the simple presence of another human being. We propose that such results are compatible with the intuition of a heterocentric reference frame linked to the addressee's perspective and built for addressing a message through pointing. A brain network for such heterocentric reference frame would encompass the right posterior STS and the pre-SMA. Interestingly, closely related areas in right pSTS and right medial prefrontal cortex were recently associated with the notion of ‘humanlikeness’ as they were more activated for playing a social game with a human partner than with robots or computer partners [71]. The heterocentric reference would presumably mark, from the subject's point of view, the notion of who is another human to be addressed, his/her own notion of the second person “you” [12]. Compared to the hypothesis of a SAM [16], this heterocentric reference goes further and places the basic nature of shared communicative attention within the domain of interpersonal relationships. This line of research places the dialog between two persons as the minimal entity to be investigated in social cognition. Testing these hypotheses from these first results in other populations might shed new light onto the bases of pathologies of social interaction such as autism or psychopathology [5], [72].

## Supporting Information

Figure S1 **Participant's trunk position in Experiment 1.** The spatial coordinates of two LEDs located on the subject's shoulders were tracked during Experiment 1 (panel a). Average orientations of the line linking the LEDs for the conditions NCP, Left CP and Right CP (panel b, c, d, respectively) at the beginning of the pointing gesture are indicated, which did not differ significantly (p>0.05).
(EPS)

Figure S2 **Participant's trunk position in Experiment 1.** The spatial coordinates of two LEDs located on the subject's shoulders were tracked during Experiment 1 (panel a). Average orientations of the line linking the LEDs for the conditions NCP, Left CP and Right CP (panel b, c, d, respectively) at the beginning of the pointing gesture are indicated, which did not differ significantly (p>0.05).
(EPS)

Table S1 **Temporal parameters of the pointing movement in Experiment 1.** RT: reaction time; cRT: corrected reaction time; ttp: time to peak of maximal velocity; dur: duration of the movement; maxvel: maximal velocity; meanvel: mean velocity; ns: not significant (p>0.05). Mean (Standard Deviation).
(DOC)

Table S2 **X coordinates of the mean trajectories in Experiment 1.** The X axis corresponds to the left-right line. For each subject and each gesture, 20 points were isolated along the trajectory, at equal time intervals (duration of the movement/20). ANOVA for x coordinates, using condition as a within-subject factor, are provided for each point. Coordinates are provided in mm; ns: not significant (p>0.05).
(DOC)

Table S3 **Z coordinates of the mean trajectories in Experiment 1.** The Z axis corresponds to the bottom-up line. For each subject and each gesture, 20 points were isolated along the trajectory, at equal time intervals (duration of the movement/20). ANOVA for z coordinates, using condition as a within-subject factor, are provided for each point. Coordinates are provided in mm; ns: not significant (p>0.05).
(DOC)

## References

[1] Liszkowski U, Carpenter M, Henning A, Striano T, Tomasello M (2004). Twelve-month-olds point to share attention and interest.. Developmental Science.

[2] Tomasello M (1999). The Cultural Origins of Human Cognition..

[3] Carpenter M, Nagell K, Tomasello M (1998). Social cognition, joint attention, and communicative competence from 9 to 15 months of age.. Monographs of the Society for the Research on Child Development.

[4] Charman T (2003). Why is joint attention a pivotal skill in autism?. Philosophical Transactions of the Royal Society of London B Biological Sciences.

[5] Hobson R, Eilan N, Hoerl C, McCormack T, Roessler J (2005). What Puts the Jointness into Joint Attention?. Joint attention: communication and other minds.

[6] Baron-Cohen S (1989). Perceptual role taking and protodeclarative pointing in autism.. British Journal of Developmental Psychology.

[7] Franco F, Eilan N, Hoerl C, McCormack T, Roessler J (2005). Infant pointing: Harlequin, Servant of Two Masters.. Joint attention: communication and other minds.

[8] Franco F, Butterworth G (1996). 'Pointing and social awareness: declaring and requesting in the second year'.. Journal of Child Language.

[9] Liszkowski U, Albrecht K, Carpenter M, Tomasello M (2008). Infants' visual and auditory communication when a partner is or is not visually attending.. Infant Behav Dev.

[10] Brooks R, Meltzoff A (2002). The Importance of Eyes: How Infants Interpret Adult Looking.. Developmental Psychology.

[11] Phillips W, Gomez JC, Baron-Cohen S, Laa V, Riviere A (1995). Treating people as objects, agents, or “subjects”: how young children with and without autism make requests.. J Child Psychol Psychiatry.

[12] Gómez JC, Eilan N, Hoerl C, McCormack T, Roessler J (2005). Joint Attention and the Notion of Subject: Insights from Apes, Normal Children, and Children with Autism.. Joint attention: communication and other minds.

[13] Charman T, Stone WL (2006). Social and communication development in autism spectrum disorders: early identification, diagnosis, and intervention..

[14] Mundy P, Sigman M, Kasari C (1990). A longitudinal study of joint attention and language development in autistic children.. Journal of Autism and Developmental Disorders.

[15] Baron-Cohen S (1995). Mindblindness: an essay on autism and theory of mind..

[16] Baron-Cohen S, Ellis BBD (2005). The Empathizing System: a revision of the 1994 model of the Mindreading System.. Origins of the Social Mind: Guilford Publications Inc.

[17] Degos J-D, Bachoud-Lévi A-C, Ergis A-M, Petrissans J-L, Cesaro P (1997). Selective inability to point to extrapersonal targets after left posterior parietal lesion: an objectivisation disorder?. Neurocase.

[18] Felician O, Ceccaldi M, Didic M, Thinus-Blanc C, Poncet M (2003). Pointing to body parts: a double dissociation study.. Neuropsychologia.

[19] Cleret de Langavant L, Trinkler I, Cesaro P, Bachoud-Levi AC (2009). Heterotopagnosia: When I point at parts of your body.. Neuropsychologia.

[20] Pick A (1922). Störung der Orienterung am eigenen Körper. Beitrag zur Lehre vom Bewusstsein des eigenen Körpers.. Psychologische Forschung.

[21] Sirigu A, Grafman J, Bressler K, Sunderland T (1991). Multiple representations contribute to the body knowledge processing; evidence from a case of autotopoagnosia.. Brain.

[22] Andersen RA, Cui H (2009). Intention, action planning, and decision making in parietal-frontal circuits.. Neuron.

[23] Soechting JF, Tillery SIH, Flanders M (1990). Transformation from head- to shoulder-centered representation of target direction in arm movements.. Journal of Cognitive Neuroscience.

[24] Flanders M, Tillery S, Soechting J (1992). Early stages in a sensorimotor transformation.. Behavioural and Brain Science.

[25] McIntyre J, Stratta F, Lacquaniti F (1997). Viewer-Centered Frame of Reference for Pointing to Memorized Targets in Three-Dimensional Space.. Journal of Neurophysiology.

[26] Carozzo M, Stratta F, McIntyre J, Lacquaniti F (2002). Cognitive allocentric representations of visual space shape pointing errors.. Experimental Brain Research.

[27] Astafiev S, Shulman G, Stanley C, Snyder A, Van Essen D (2003). M. Functional organization of human intraparietal and frontal cortex for attending, looking and pointing.. Journal of Neuroscience.

[28] Grafton S, Fagg A, Woods R, Arbib M (1996). Functional anatomy of pointing and grasping in humans.. Cerebral Cortex.

[29] Simon O, Khérif F, Flandin G, Poline J, Riviere D (2004). Automatized clustering and functional geometry of human parietofrontal networks for language, space, and number.. Neuroimage.

[30] Simon O, Mangin JF, Cohen L, Le Bihan D, Dehaene S (2002). Topographical layout of hand, eye, calculation, and language-related areas of the huaman parietal lobe.. Neuron.

[31] Perrett DI, Oram MW, Harries MH, Bevan R, Hietanen JK (1991). Viewer-centred and object-centred coding of heads in the macaque temporal cortex.. Exp Brain Res.

[32] Emery NJ (2000). The eyes have it: the neuroethology, function and evolution of social gaze.. Neurosci Biobehav Rev.

[33] Jellema T, Maassen G, Perrett DI (2004). Single cell integration of animate form, motion and location in the superior temporal cortex of the macaque monkey.. Cereb Cortex.

[34] Puce A, Allison T, Bentin S, Gore J, McCarthy G (1998). Temporal cortex activation in humans viewing eye and mouth movements.. Journal of Neuroscience.

[35] Wicker B, Michel F, Henaff MA, Decety J (1998). Brain regions involved in the perception of gaze: a PET study.. Neuroimage.

[36] Pelphrey K, Singerman J, Allison T, McCarthy G (2003). Brain activation evoked by perception of gaze shifts: the influence of context.. Neuropsychologia.

[37] Zilbovicius M, Meresse I, Chabane N, Brunelle F, Samson Y (2006). Autism, the superior temporal sulcus and social perception.. Trends Neurosci.

[38] Bookstein FL (1992). Error analysis, regression and coordinate systems (commentary to Flanders et al.).. Behavioral and Brain Sciences.

[39] Gordon J, Ghilardi M, Ghez C (1994). Accuracy of planar reaching movements. I. Independence of direction and extent variability.. Exp Brain Res.

[40] Lacquaniti F, Boller F, Grafman J (1997). Frames of reference in sensorimotor coordination.. Handbook of Neuropsychology.

[41] Oldfield RC (1971). The assessment and analysis of handedness: the Edinburgh Inventory.. Neuropsychologia.

[42] Pallier C, Dupoux E, Jeannin X (1997). Expe: an expandable programming language for on-line psychological experiments.. Behavior Research Methods, Instruments and Computers.

[43] Cook RD, Weisberg S (1982). Residuals and Influence in Regression..

[44] Ihaka R, Gentleman R (1996). R: A language for data analysis and graphics.. Journal of Computational and Graphical Statistics.

[45] Vindras P, Viviani P (1998). Frames of reference and control parameters in visuomanual pointing.. Journal of Experimental Psychology: Human Perception and Performance.

[46] Howard LA, Tipper SP (1997). Hand deviations away from visual cues: indirect evidence for inhibition.. Exp Brain Res.

[47] Diedrichsen J, Werner S, Schmidt T, Trommershaüser J (2004). Immediate spatial distortions of pointing movements induced by visual landmarks.. Percept Psychophys.

[48] Trommershaüser J, Maloney L, Landy M (2003). Statistical decision theory and the selection of rapid, goal-directed movements.. Journal of Optical Society American A Optics and Image Science Vission.

[49] Frith U, de Vignemont F (2005). Egocentrism, allocentrism, and Asperger syndrome.. Conscious Cogn.

[50] Langdon R, Coltheart M (2001). Visual perspective-taking and schizotypy: evidence for a simulation-based account of mentalizing in normal adults.. Cognition.

[51] Decety J, Grezes J (1999). Neural mechanisms subserving the perception of human actions.. Trends in Cognitive Sciences.

[52] Allison T, Puce A, McCarthy G (2000). Social perception from visual cues: role of the STS region.. Trends in Cognitive Sciences.

[53] Pelphrey K, Morris J, McCarthy G (2004). Grasping the intentions of others: the perceived intentionality of an action influences activity in the superior temporal sulcus during social perception.. Journal of Cognitive Neuroscience.

[54] Rizzolatti G, Fogassi L, Gallese V (2002). Motor and cognitive functions of the ventral premotor cortex.. Current Opinion in Neurobiology.

[55] Saxe R, Kanwisher N (2003). People thinking about thinking people. The role of the temporo-parietal junction in “theory of mind”.. Neuroimage.

[56] Kriegstein KV, Giraud AL (2004). Distinct functional substrates along the right superior temporal sulcus for the processing of voices.. NeuroImage.

[57] Chan AW, Peelen MV, Downing PE (2004). The effect of viewpoint on body representation in the extrastriate body area.. NeuroReport.

[58] Saxe R, Jamal N, Powell L (2006). My Body or Yours? The Effect of Visual Perspective on Cortical Body Representations.. Cerebral Cortex.

[59] Abraham A, Werning M, Rakoczy H, von Cramon DY, Schubotz RI (2008). Minds, persons, and space: an fMRI investigation into the relational complexity of higher-order intentionality.. Conscious Cogn.

[60] David N, Bewernick BH, Cohen MX, Newen A, Lux S (2006). Neural representations of self versus other: visual-spatial perspective taking and agency in a virtual ball-tossing game.. J Cogn Neurosci.

[61] Ramnani N, Miall RC (2004). A system in the human brain for predicting the actions of others.. Nature neuroscience.

[62] Blanke O, Landis T, Spinelli L, Seeck M (2004). Out-of-body experience and autoscopy of neurological origin.. Brain.

[63] Spence SA (2002). Alien motor phenomena: a window on to agency.. Cogn Neuropsychiatry.

[64] Spence SA, Brooks DJ, Hirsch SR, Liddle PF, Meehan J (1997). A PET study of voluntary movement in schizophrenic patients experiencing passivity phenomena (delusions of alien control).. Brain.

[65] Ganesan V, Hunter MD, Spence SA (2005). Schneiderian first-rank symptoms and right parietal hyperactivation: a replication using FMRI.. Am J Psychiatry.

[66] Nachev P, Kennard C, Husain M (2008). Functional role of the supplementary and pre-supplementary motor areas.. Nature reviews Neuroscience.

[67] Jenkins IH, Jahanshahi M, Jueptner M, Passingham RE, Brooks DJ (2000). Self-initiated versus externally triggered movements. II. The effect of movement predictability on regional cerebral blood flow.. Brain.

[68] Jahanshahi M, Jenkins IH, Brown RG, Marsden CD, Passingham RE (1995). Self-initiated versus externally triggered movements. I. An investigation using measurement of regional cerebral blood flow with PET and movement-related potentials in normal and Parkinson's disease subjects.. Brain.

[69] Nachev P, Rees G, Parton A, Kennard C, Husain M (2005). Volition and conflict in human medial frontal cortex.. Curr Biol.

[70] Henderson LM, Yoder PJ, Yale ME, McDuffie A (2002). Getting the point: electrophysiological correlates of protodeclarative pointing.. Int J Dev Neurosci.

[71] Krach S, Hegel F, Wrede B, Sagerer G, Binkofski F (2008). Can Machines Think? Interaction and Perspective Taking with Robots Investigated via fMRI.. PLoS One.

[72] Gómez JC (1996). Second-person intentional relations and the evolution of social understanding.. Behavioural and Brain Sciences.

