# Supplementary material for: Behavioral and Neural Correlates of Communication via Pointing
Source: PLoS One. 2011 Mar 15;6(3):e17719. doi: 10.1371/journal.pone.0017719 (PMC3057969; doi:10.1371/journal.pone.0017719)
Supplement: Table S1 — Temporal parameters of the pointing movement in Experiment 1. RT: reaction time; cRT: corrected reaction time; ttp: time to peak of maximal velocity; dur: duration of the movement; maxvel: maximal velocity; meanvel: mean velocity; ns: not significant (p>0.05). Mean (Standard Deviation). (DOC) [file pone.0017719.s003.doc]

|  | Left CP | Right CP | NCP | Statistics |
| --- | --- | --- | --- | --- |
| RT (ms) | 683.3 (77.5) | 685.4 (88.3) | 686.1 (81.9) | ns |
| cRT (ms) | 709.0 (74.4) | 708.9 (82.6) | 707.9 (79.6) | ns |
| dur(ms) | 688.7 (84.6) | 685.6 (89.1) | 679.0 (85.6) | ns |
| ttp (ms) | 289.1 (46.6) | 286.9 (50.7) | 283.0 (50.8) | ns |
| maxvel (m/s) | 1.36 (0.16) | 1.35 (0.18) | 1.34 (0.18) | ns |
| meanvel (m/s) | 0.63 (0.088) | 0.63 (0.093) | 0.64 (0.087) | ns |
