# Supplementary material for: Behavioral and Neural Correlates of Communication via Pointing
Source: PLoS One. 2011 Mar 15;6(3):e17719. doi: 10.1371/journal.pone.0017719 (PMC3057969; doi:10.1371/journal.pone.0017719)
Supplement: Table S2 — X coordinates of the mean trajectories in Experiment 1. The X axis corresponds to the left-right line. For each subject and each gesture, 20 points were isolated along the trajectory, at equal time intervals (duration of the movement/20). ANOVA for x coordinates, using condition as a within-subject factor, are provided for each point. Coordinates are provided in mm; ns: not significant (p>0.05). (DOC) [file pone.0017719.s004.doc]

|  | Left CP | Right CP | NCP | ANOVA |
| --- | --- | --- | --- | --- |
| X1 | 3.2 | 2.9 | 3.2 | ns |
| X2 | 5.0 | 4.7 | 5.2 | ns |
| X3 | 11.3 | 11.2 | 11.8 | ns |
| X4 | 24.7 | 24.7 | 25.3 | ns |
| X5 | 46.3 | 46.1 | 46.6 | ns |
| X6 | 75.4 | 75.0 | 75.2 | ns |
| X7 | 110.8 | 109.9 | 109.6 | ns |
| X8 | 150.0 | 147.9 | 147.4 | ns |
| X9 | 190.1 | 186.8 | 186.0 | ns |
| X10 | 228.1 | 224.0 | 222.9 | ns |
| X11 | 262.1 | 257.4 | 256.2 | ns |
| X12 | 290.8 | 286.0 | 284.7 | p = 0.025 |
| X13 | 313.3 | 308.7 | 307.6 | p = 0.012 |
| X14 | 329.6 | 325.7 | 324.8 | p = 0.009 |
| X15 | 340.9 | 337.9 | 337.1 | p = 0.014 |
| X16 | 348.4 | 346.0 | 345.3 | p = 0.022 |
| X17 | 352.9 | 351.1 | 350.5 | p = 0.028 |
| X18 | 355.2 | 353.8 | 353.2 | p = 0.037 |
| X19 | 355.8 | 354.5 | 354.0 | p = 0.03 |
| X20 | 355.9 | 354.7 | 354.4 | p = 0.021 |
