# Supplementary material for: Behavioral and Neural Correlates of Communication via Pointing
Source: PLoS One. 2011 Mar 15;6(3):e17719. doi: 10.1371/journal.pone.0017719 (PMC3057969; doi:10.1371/journal.pone.0017719)
Supplement: Table S3 — Z coordinates of the mean trajectories in Experiment 1. The Z axis corresponds to the bottom-up line. For each subject and each gesture, 20 points were isolated along the trajectory, at equal time intervals (duration of the movement/20). ANOVA for z coordinates, using condition as a within-subject factor, are provided for each point. Coordinates are provided in mm; ns: not significant (p>0.05). (DOC) [file pone.0017719.s005.doc]

|  | Left CP | Right CP | NCP | ANOVA |
| --- | --- | --- | --- | --- |
| Z1 | 2.0 | 1.8 | 1.7 | ns |
| Z2 | 6.0 | 5.7 | 5.3 | ns |
| Z3 | 16.6 | 15.6 | 14.6 | p = 0.049 |
| Z4 | 30.6 | 28.7 | 27.0 | p = 0.014 |
| Z5 | 43.3 | 40.6 | 38.3 | p = 0.006 |
| Z6 | 50.8 | 47.7 | 44.6 | p = 0.009 |
| Z7 | 51.5 | 48.2 | 44.5 | p = 0.024 |
| Z8 | 45.7 | 42.4 | 38.0 | ns |
| Z9 | 33.7 | 30.6 | 25.6 | ns |
| Z10 | 16.3 | 13.5 | 8.4 | ns |
| Z11 | -4.9 | -6.8 | -11.8 | ns |
| Z12 | -27.7 | -28.7 | -32.9 | ns |
| Z13 | -49.7 | -50.0 | -53.2 | ns |
| Z14 | -69.4 | -69.2 | -71.3 | ns |
| Z15 | -85.9 | -85.4 | -86.7 | ns |
| Z16 | -99.0 | -98.5 | -99.4 | ns |
| Z17 | -109.5 | -109.2 | -109.7 | ns |
| Z18 | -118.4 | -118.3 | -118.5 | ns |
| Z19 | -126.1 | -126.2 | -126.2 | ns |
| Z20 | -130.2 | -130.3 | -130.2 | ns |
